# Factors Associated with Total Laryngectomy Utilization in Patients with cT4a Laryngeal Cancer

**DOI:** 10.3390/cancers15225447

**Published:** 2023-11-16

**Authors:** Alex R. Ritter, Vedat O. Yildiz, Nischal Koirala, Sujith Baliga, Emile Gogineni, David J. Konieczkowski, John Grecula, Dukagjin M. Blakaj, Sachin R. Jhawar, Kyle K. VanKoevering, Darrion Mitchell

**Affiliations:** 1 Department of Radiation Oncology, The Ohio State University Wexner Medical Center, 410 W 10th Ave., Columbus, OH 43210, USA; 2 Department of Biomedical Informatics, Center for Biostatistics, Ohio State University, 1800 Cannon Dr., Columbus, OH 43210, USA; 3 Department of Otolaryngology-Head and Neck Surgery, The Ohio State University Wexner Medical Center, 410 W 10th Ave., Columbus, OH 43210, USA

**Keywords:** laryngeal cancer, total laryngectomy, radiotherapy, practice patterns

## Abstract

**Simple Summary:**

Total laryngectomy (TL) with post-operative radiotherapy (PORT) is recommended for patients with clinically advanced (cT4a) laryngeal cancer and is associated with superior outcomes compared to non-operative treatment with chemoradiation. Despite this, many patients with a diagnosis of laryngeal cancer do not receive TL as part of their treatment course, which places them at risk for inferior oncologic outcomes, and the factors associated with the utilization of TL in this setting remain poorly understood. Thus, the aims of this observational cohort study utilizing the National Cancer Database (NCDB) were both to elucidate the patient and treatment facility characteristics associated with TL utilization in patients with cT4a laryngeal cancer and to evaluate how its usage has changes over time.

**Abstract:**

Background: Despite recommendations for upfront total laryngectomy (TL), many patients with cT4a laryngeal cancer (LC) instead undergo definitive chemoradiation, which is associated with inferior survival. Sociodemographic and oncologic characteristics associated with TL utilization in this population are understudied. Methods: This retrospective cohort study utilized hospital registry data from the National Cancer Database to analyze patients diagnosed with cT4a LC from 2004 to 2017. Patients were stratified by receipt of TL, and patient and facility characteristics were compared between the two groups. Logistic regression analyses and Cox proportional hazards methodology were performed to determine variables associated with receipt of TL and with overall survival (OS), respectively. OS was estimated using the Kaplan–Meier method and compared between treatment groups using log-rank testing. TL usage over time was assessed. Results: There were 11,149 patients identified. TL utilization increased from 36% in 2004 to 55% in 2017. Treatment at an academic/research program (OR 3.06) or integrated network cancer program (OR 1.50), male sex (OR 1.19), and Medicaid insurance (OR 1.31) were associated with increased likelihood of undergoing TL on multivariate analysis (MVA), whereas age > 61 (OR 0.81), Charlson–Deyo comorbidity score ≥ 3 (OR 0.74), and clinically positive regional nodes (OR 0.78 [cN1], OR 0.67 [cN2], OR 0.21 [cN3]) were associated with decreased likelihood. Those undergoing TL with post-operative radiotherapy (+/− chemotherapy) had better survival than those receiving chemoradiation (median OS 121 vs. 97 months; *p* = 0.003), and TL + PORT was associated with lower risk of death compared to chemoradiation on MVA (HR 0.72; *p* = 0.024). Conclusions: Usage of TL for cT4a LC is increasing over time but remains below 60%. Patients seeking care at academic/research centers are significantly more likely to undergo TL, highlighting the importance of decreasing barriers to accessing these centers. Increased focus should be placed on understanding and addressing the additional patient-, physician-, and system-level factors that lead to decreased utilization of surgery.

## 1. Introduction

Laryngeal cancer accounts for 12,470 cases and 3820 deaths annually in the United States [1]. Incidence peaks in the seventh decade of life, and there is a significant male predominance [2]. Risk factors for laryngeal cancer include smoking, alcohol, and environmental exposures such as chlorinated solvents [3].

Standard treatment for early-stage laryngeal cancer typically consists of larynx-sparing surgery or radiotherapy alone. Total laryngectomy (TL) with neck dissection followed by post-operative radiotherapy (PORT) remains a standard treatment option for patients with locally advanced disease; the results of two landmark randomized trials helped establish organ preservation with definitive-intent chemoradiation as an alternative for many of these patients. The Veterans’ Administration Laryngeal Cancer Study Group (VALCSG) trial demonstrated that induction chemotherapy followed by radiation for those with treatment response offered survival equivalent to upfront laryngectomy followed by PORT [4]. The subsequent Radiation Therapy Oncology Group (RTOG) 91-11 trial demonstrated the superiority of concurrent chemoradiation with regard to larynx preservation compared to sequential chemotherapy followed by radiation and to radiation alone [5]. 

Avoidance of TL certainly has benefits, as TL can have significant negative impact on swallowing, speech, and respiration [6,7], and patients undergoing TL with PORT are more likely than those undergoing chemoradiation to experience smell and taste disturbances, increased usage of painkillers, and coughing [8]; moreover, psychological well-being can also be negatively impacted following TL, with stress, anxiety, and depression often reported [9]. The rates of laryngeal preservation were 64% at 2 years on the VALCSG trial [4] and 81.7% at 10 years in RTOG 91-11 [5], suggesting that many patients with advanced laryngeal cancer can successfully avoid TL. One question that arises, however, is the applicability and appropriateness of organ preservation in patients with cT4a disease (defined as tumor invasion beyond the outer cortex of the thyroid cartilage and/or the tissues beyond the larynx) in particular. These patients required salvage laryngectomy in 56% of cases following attempted organ preservation in the VALCSG trial, leading to the exclusion of “high-volume T4 primaries” (defined as >1 cm of base of tongue invasion or penetration through cartilage) in RTOG 91-11, with only 10% of total patients in this trial having T4 disease of any kind. 

Due to the relatively poor outcomes and underrepresentation of cT4a tumors in these trials, current guidelines from the National Comprehensive Cancer Network (NCCN) for the management of cT4a laryngeal cancer recommend against organ preservation with chemoradiation, aside from select patients who decline surgery [10]. Despite this, multiple national database analyses have demonstrated an increase in organ preservation with compensatory decline in TL from the 1980s to the 2000s for cT4a laryngeal cancer [11,12]. During this same period, survival rates for laryngeal cancer declined, whereas survival rates for all other cancer types improved [13]. This curious observation led to speculation that the ongoing substitution of chemoradiation for TL, particularly in patients with cT4a cancer, was driving declining outcomes [11,14]. 

Numerous disparities relating to race, ethnicity, and socioeconomic status in cancer have been well documented, both overall [1] and within head and neck cancer specifically [15,16,17,18,19,20,21]. These disparities exist regarding not only outcomes, but treatment strategies as well. For example, numerous studies have demonstrated that Black patients are significantly less likely than White patients to undergo surgery for oropharyngeal cancer [18], lung cancer [22,23], and colorectal cancer [24,25,26], even after controlling for other sociodemographic and disease characteristics. Other sociodemographic factors such as income [27,28] and insurance status [28] have been associated with decreased likelihood of surgical utilization in other cancer types, but the relationship between these factors and the use of TL in patients with laryngeal cancer is less understood. Given the importance of surgery for cT4a laryngeal cancer, we sought to characterize the sociodemographic, oncologic, and clinical factors associated with TL usage in this population and to assess their relationship with survival outcomes.

## 2. Materials and Methods

### 2.1. Data Source and Patient Selection

The National Cancer Database (NCDB), a joint project of the Commission on Cancer (CoC) of the American College of Surgeons and the American Cancer Society, is a national clinical oncology hospital registry database sourced from more than 1500 CoC-accredited facilities in the United States and Puerto Rico, which tracks patient demographics, disease characteristics, treatments, and overall survival (OS) for patients diagnosed with cancer. The NCDB was queried to identify patients ≥ 18 years of age diagnosed with laryngeal cancer between 2004 and 2017 (using the International Classification of Diseases for Oncology, Third Edition [ICD-O-3]; topography codes C32.0–C32.9) and staged with cT4a disease (according to the American Joint Committee on Cancer [AJCC] 6th or 7th edition, depending on year of diagnosis). Because the utilized data were deidentified and obtained from a preexisting, publicly accessible database, the study was exempt from review by our Institutional Review Board. Patients with metastatic disease, missing vital status information, unknown treatment status, and incomplete staging were excluded from analysis. TL was defined using the site-specific Surveillance, Epidemiology, and End Results (SEER) surgical codes for “total or radical laryngectomy, NOS” (code 40), “total laryngectomy” (code 41), “radical laryngectomy” (code 42), and “pharyngolaryngectomy” (code 50). Disease characteristics, treatment facility characteristics, and patient demographic information were extracted. 

### 2.2. Statistical Analysis

Clinical, oncologic, and demographic variables were summarized by frequency and percent for categorical variables, by mean and standard deviation for normally distributed variables, and by median and interquartile range (IQR) for abnormally distributed variables. Categorical variables were compared between patients undergoing TL (either in the upfront or salvage setting) and those who did not, using the Chi-Square or Fisher’s Exact test, while continuous variables were compared between the two groups using the unpaired *t*-test. Univariate (UVA) and multivariate (MVA) logistic regression analyses were performed to determine demographic variables associated with the receipt of TL; pathologic disease characteristics were not included as these factors would not have been known prior to the decision to undergo surgery. Additional UVA and MVA using Cox proportional hazards methodology were performed to determine the demographic and clinical variables associated with OS. In both cases, variables with *p* < 0.20 on UVA were included in MVA, apart from facility location and lymphovascular space invasion, which were excluded from the MVA for OS due to small sample size. We tested the proportionality assumption including covariate interactions with time as a predictor in the Cox model; the proportionality assumption was not violated. OS was estimated using the Kaplan–Meier method, with log-rank testing utilized to compare survival between patients with different clinical and demographic characteristics. Survival was compared between the following three groups based on treatment received, with Bonferroni corrections used for pairwise comparisons: TL alone (without radiation), upfront TL and PORT to 6000 cGy or more (with or without chemotherapy), and chemoradiation (receiving ≥ 6996 cGy radiation in addition to chemotherapy without undergoing surgery). The usage of TL over time and reasons for TL not being performed were also assessed.

## 3. Results

After applying inclusion and exclusion criteria, 11,149 patients were available for analysis, of whom 5049 (45%) underwent TL and 6100 (55%) did not. A summary of demographic and disease characteristics cross-tabulated by receipt of TL is provided in Table 1. Patients who underwent TL were more likely to be younger (mean age 61 vs. 63 years; *p* < 0.001), male (83% vs. 79%; *p* < 0.001), non-Hispanic (91% vs. 89%; *p* = 0.001), live in non-metropolitan counties (22% vs. 19%; *p* < 0.001), receive treatment at an academic/research institution (64% vs. 40%; *p* < 0.001), travel more than 30 miles for treatment (33% vs. 19%; *p* < 0.001), have lower clinical regional nodal disease burden (63% cN+ vs. 52% cN0; *p* < 0.001), and have lower comorbid disease burden (62% for Charlson–Deyo score 0 vs. 65% for score > 0; *p* < 0.001). Both groups had similar compositions of privately insured (25% each) and uninsured (9% vs. 10%) patients. Racial composition was not significantly different between groups (79% White and 19% Black patients in the TL group compared to 77% White and 20% black in the non-TL group; *p* = 0.243).

Clinicopathologic and demographic factors associated with the usage of TL on UVA are described in Supplementary Table S1. Factors that remained significant for increased likelihood of TL usage on MVA (Table 2) included male sex (OR 1.19; *p* = 0.001), Medicaid insurance compared to private insurance/managed care (OR 1.31; *p* < 0.001), 3rd quartile income compared to 1st (OR 1.20; *p* = 0.045), treatment at an academic/research institution (OR 3.06; *p* < 0.001) or integrated network cancer program (OR 1.50; *p* = 0.003) compared to a community institution, travel from 5 to 30 miles (OR 1.35; *p* < 0.001) and >30 miles (OR 2.55; *p* < 0.001) for treatment compared to <5 miles, and diagnosis in 2011–2017 compared to 2004–2010 (OR 1.40; *p* < 0.001). Age > 61 (OR 0.81; *p* < 0.001), urban county of residence (OR 0.82; *p* = 0.019) compared to metro, Charlson–Deyo score ≥ 3 (OR 0.74; *p* = 0.031) compared to 0, and clinically positive regional nodes (OR 0.78 for cN1, OR 0.67 for cN2, and OR 0.21 for cN3; *p* < 0.001 for all) were factors associated with decreased likelihood of TL utilization on MVA.

**Figure 1:**
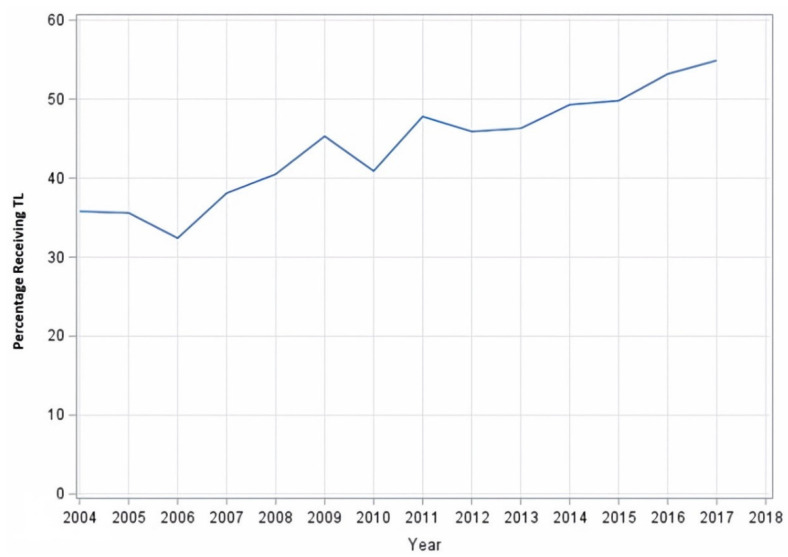
Total Laryngectomy Utilization Trend. Percentage of T4a patients receiving total laryngectomy (TL) over time.

The utilization of TL for disease increased over time, from 36% of patients in 2004 to 55% in 2017, as shown in Figure 1. A summary of provided reasons for lack of surgery are summarized in Supplementary Table S2. Among patients not receiving TL, the most common reason the surgery was not being performed was that it was not recommended by the physician (85.7%). In 5.0% of cases, TL was felt to be contraindicated due to patient risk factors such as comorbid conditions or advanced age.

Clinicopathologic and socioeconomic factors associated with increased risk of death on UVA are described in Supplementary Table S3. As shown in Table 3, factors that remained significant for increased risk of death on MVA included age > 61 (HR 1.32; *p* < 0.001), male sex (HR 1.13; *p* = 0.002), lack of private insurance (HR 1.32 for Medicaid [*p* < 0.001], HR 1.52 for Medicare [*p* < 0.001], HR 1.43 for other governmental insurance [*p* = 0.001], and HR 1.26 for unknown insurance status [*p* = 0.016]), increasing Charlson–Deyo score (HR 1.11 for 1, HR 1.46 for 2, and HR 1.41 for 3; *p* < 0.001 for all), and clinically positive regional nodes (HR 1.10 for cN1 [*p* = 0.014], HR 1.41 for cN2 [*p* < 0.001], and HR 1.77 for cN3 [*p* < 0.001]). Diagnosis in years 2011–2017 (HR 0.94; *p* = 0.049), receipt of TL (HR 0.50; *p* < 0.001), receipt of radiation (HR 0.49; *p* < 0.001), and HPV-positivity (HR 0.68; *p* = 0.002) were associated with decreased risk of death on MVA.

**Figure 2:**
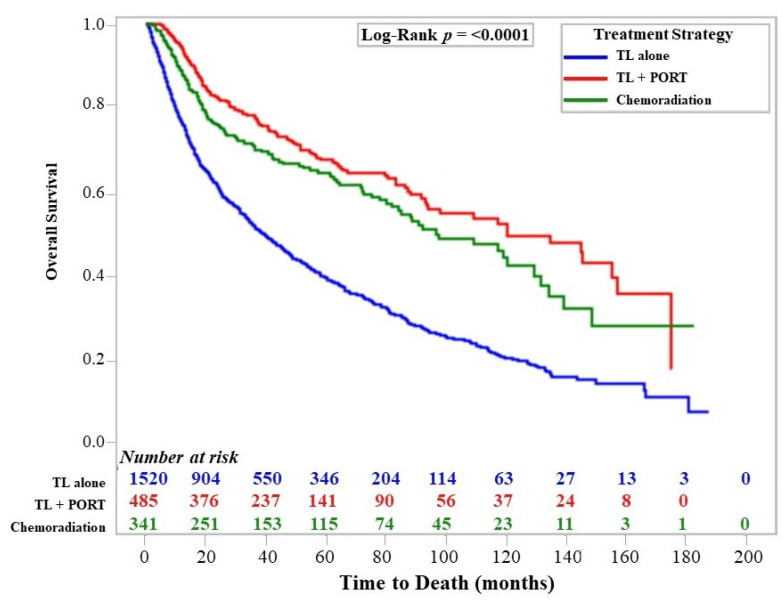
Overall Survival by Treatment Strategy. Kaplan–Meier estimates of overall survival for patients by treatment strategy. TL = total laryngectomy; PORT = post-operative radiation therapy.

When comparing survival by treatment group, those undergoing TL with PORT had significantly better OS than those receiving chemoradiation (median OS 121 vs. 97 months; *p* = 0.003) and those receiving TL alone (median OS 121 vs. 39 months; *p* < 0.001), as shown in Figure 2. When a repeat Cox regression analysis with the addition of a treatment group variable consisting of the three treatment strategies was performed, chemoradiation (HR 1.39, 95% CI 1.04–1.87; *p* = 0.024) and TL alone (HR 2.67, 95% CI 2.15–3.31; *p* < 0.001) were both associated with increased risk of death on multivariate analysis relative to TL + PORT.

## 4. Discussion

This large observational cohort study showed that only 45% of patients with cT4a laryngeal cancer underwent TL during the period evaluated; however, rates of surgery utilization in this population did increase over time from 36% in 2004 to 55% in 2017. This contrasts with trends noted throughout the 1980s–2000s, during which surgical management of cT4a disease appeared to be decreasing in favor of organ preservation. One NCDB analysis demonstrated an increase in the usage of chemoradiation for advanced laryngeal cancer from 2.3% in 1985 to 13.2% in 2001, with a corresponding decline in the usage of surgery [11]. An additional NCDB analysis evaluating treatment trends for advanced laryngeal cancer from 1985 to 2007 demonstrated an increase in usage of chemoradiation from 7% to 45% and a decline in the usage of total laryngectomy from 42% to 32% during that time period [12]. Among these patients, those treated with chemoradiation had significantly higher risk of death on multivariate analysis than those treated with laryngectomy (HR 1.13; *p* < 0.05). One potential explanation for the reversal in trend noted in our study is the concomitant increase in patients treated at academic programs, a treatment factor associated with increased likelihood of TL utilization, over time (from 45.3% in 2004 to 56.7% in 2017). Other possible reasons include changes in provider recommendations and referral patterns following the publishing of the aforementioned studies, which highlight concern over the omission of TL, advances in electrolarynx and other voice-assistive devices, and improvements in other quality of life measures that may make the decision to undergo TL more palatable for patients.

One of the largest predictors of TL utilization was treatment at an academic/research program (OR 3.06). The rate of TL utilization for patients treated at academic/research programs in this cohort was 57%, compared to only 33% at non-academic/research programs. The NCDB categorizes each treating facility based upon its classification by the CoC Accreditation program, which distinguishes academic programs as those participating in graduate medical education in addition to reporting more than 500 new cancer diagnoses annually, compared to community cancer programs, which report between 100 and 500 new cancer diagnoses each year. These findings are consistent with previous studies that have also demonstrated increased likelihood of undergoing oncologic surgery at high-volume centers [29,30,31,32]. Many studies have also demonstrated superior outcomes in patients receiving cancer treatment at high-volume centers compared to low-volume [32,33,34,35]. Barriers to seeking cancer treatment, including surgery, at high-volume centers are multifactorial and complex but, among them, are the decentralization of oncologic surgeries in the United States and limitations placed on coverage by insurance plans [36,37,38,39,40]. Therefore, public policy solutions that address these access barriers would be beneficial moving forward.

Increasing clinical nodal burden was found to negatively correlate with likelihood of surgery, as 52% of cN0 patients underwent TL compared to 43% of cN1, 41% of cN2, and 18% of cN3. These findings are consistent with other studies that have also demonstrated higher rates of chemoradiation utilization relative to TL in patients with increasing regional nodal burden [41,42]. This is despite the NCCN’s recommendation for TL for non-metastatic cT4a laryngeal cancer patients regardless of regional nodal burden. Age > 61 (OR 0.81) and Charlson–Deyo score ≥ 3 (OR 0.74) were also associated with decreased surgical utilization in our study, suggesting potential concern for increased perioperative morbidity/mortality in these populations, although many experts agree that age alone should not be a contraindication for oncologic surgery [43,44,45,46]. It should also be noted that, even among those with a Charlson–Deyo score of 0, TL utilization in this study was only 44%; contraindication due to patient risk factors such as advanced age or comorbidities was listed as the reason for no surgery in only 5.0%. This suggests that even young and healthy patients with cT4a laryngeal cancer are frequently not recommended TL as part of their care.

In contrast, the additional sociodemographic characteristics evaluated were largely not correlated with TL utilization on MVA, apart from increased utilization in those with Medicaid insurance (OR 1.31) and in male patients (OR 1.19); however, insurance through Medicaid (HR 1.32), Medicare (HR 1.52), and other governmental sources (HR 1.43) were also associated with increased risk of death on MVA, suggesting that other risk factors are more prevalent in these cohorts and are driving the inferior survival outcomes. Our study did demonstrate that TL followed by PORT was associated with a modest but significant benefit in overall survival compared to non-operative management with chemoradiation, which is consistent with several other retrospective studies demonstrating a survival advantage with TL [41,42,47,48,49,50]. Of note, those receiving TL alone without PORT had significantly worse median overall survival than those receiving TL + PORT (39 vs. 121 months; *p* < 0.001) or those receiving definitive-dose chemoradiation (39 vs. 97 months; *p* < 0.001). This justifies PORT as the standard of care following TL in locally advanced laryngeal cancer and highlights the survival benefit of guideline-based multimodality therapy in these cases.

An interesting positive prognostic factor for overall survival in our study was tumor HPV-positivity (HR 0.68, 95% CI 0.53–0.86; *p* = 0.002). In oropharyngeal cancer, HPV-positivity is a positive prognostic and predictive factor, and represents a biologically distinct disease process, but its significance in other cancers of the head and neck has not been clearly established [51,52]. It is possible that the apparent prognostic significance of HPV-positivity in laryngeal cancer suggested by our study is simply related to the miscoding of bulky oropharyngeal primaries with extension into the larynx as laryngeal primaries. Our findings do, nonetheless, suggest that HPV testing of laryngeal tumors may need further consideration for its potential prognostic significance, particularly as the interpretation of the available data is limited given that the routine HPV testing of non-oropharyngeal head and neck cancer subsites is not standard of care.

Additional limitations of this study are similar to many retrospective analyses involving national databases. Patients were included on the bases of their *ICD* and surgical codes, so potential miscoding could lead to data inaccuracy. The only disease-specific endpoint available in the NCDB is overall survival, so evaluation of other oncologic endpoints such as progression-free survival or local control are not possible. While the NCDB captures more than 70% of cancer cases in the United States, it is possible that, due to the data coming from Commission on Cancer-accredited facilities, selection bias may be introduced. A final potential limitation is that the TL cohort also included those who received TL in the salvage setting, which represents a distinct clinical circumstance and thus may reflect different predisposing factors than those associated with upfront utilization; however, the percentage who underwent salvage TL in this study’s cohort was quite low (only 3% of all patients who underwent TL) and would therefore be unlikely to skew the results.

## 5. Conclusions

TL utilization for cT4a laryngeal cancer increased from 2004 to 2017 but remains below 60% despite consensus recommendations and a demonstrated survival benefit to upfront surgical management in this setting. Patients receiving care at an academic/research program are significantly more likely to receive TL than those at a community center, which highlights the importance of minimizing barriers to accessing care at these centers. Further work is warranted on understanding and addressing the patient-, physician-, and system-level factors that lead to the decreased utilization of surgery as well as the additional socioeconomic factors that may compound them. All patients need to be educated on the potential risks and benefits of the competing therapeutic modalities, ideally in a multidisciplinary setting.

## Figures and Tables

**Table 1:** Baseline Characteristics.

	Variable	No Total Laryngectomy (*n* = 6100)	Total Laryngectomy (*n* = 5049)	Total (*n* = 11,149)	*p*-Value
Facility Type	Community Cancer Program	374 (6%)	146 (3%)	520 (5%)	<0.001
	Comprehensive Community Cancer Program	2143 (35%)	978 (20%)	3121 (28%)	
	Academic/Research Program	2423 (40%)	3195 (64%)	5618 (51%)	
	Integrated Network Cancer Program	1107 (18%)	661 (13%)	1768 (16%)	
Facility Location	New England	309 (5%)	176 (4%)	485 (4%)	<0.001
	Middle Atlantic	955 (16%)	683 (14%)	1638 (15%)	
	South Atlantic	1498 (25%)	1229 (25%)	2727 (25%)	
	East North Central	1166 (19%)	990 (20%)	2156 (20%)	
	East South Central	599 (10%)	395 (8%)	994 (9%)	
	West North Central	399 (7%)	425 (9%)	824 (7%)	
	West South Central	506 (8%)	531 (11%)	1037 (9%)	
	Mountain	209 (3%)	140 (3%)	349 (3%)	
	Pacific	406 (7%)	411 (8%)	817 (7%)	
Age at Diagnosis	Mean (SD)	63 (11.1)	61 (10.1)	62 (10.7)	<0.001
Sex	Male	4808 (79%)	4176 (83%)	8984 (81%)	<0.001
	Female	1292 (21%)	873 (17%)	2165 (19%)	
Race	White	4726 (77%)	3976 (79%)	8702 (78%)	0.243
	Black	1212 (20%)	953 (19%)	2165 (19%)	
	Other/Unknown	162 (3%)	120 (2%)	282 (3%)	
Ethnicity	Non-Hispanic	5450 (89%)	4581 (91%)	10,031 (90%)	0.001
	Hispanic	367 (6%)	301 (6%)	668 (6%)	
	Unknown	283 (5%)	167 (3%)	450 (4%)	
Insurance Type	Private Insurance/Managed Care	1507 (25%)	1276 (25%)	2783 (25%)	<0.001
	Not Insured	583 (10%)	453 (9%)	1036 (9%)	
	Medicaid	1132 (19%)	1225 (24%)	2357 (21%)	
	Medicare	2589 (42%)	1904 (38%)	4493 (40%)	
	Other Government	136 (2%)	96 (2%)	232 (2%)	
	Unknown	153 (3%)	95 (2%)	248 (2%)	
Percentage with No High School Degree	≥21.0%	1521 (27%)	1250 (28%)	2771 (27%)	0.107
	13.0–20.9%	1821 (32%)	1501 (33%)	3322 (33%)	
	7.0–12.9%	1595 (28%)	1222 (27%)	2817 (28%)	
	<7.0%	762 (13%)	547 (12%)	1309 (13%)	
Annual Income	1st Quartile	1508 (27%)	1086 (25%)	2594 (26%)	0.010
	2nd Quartile	1564 (28%)	1271 (29%)	2835 (28%)	
	3rd Quartile	1211 (22%)	1053 (24%)	2264 (23%)	
	4th Quartile	1290 (23%)	1006 (23%)	2296 (23%)	
County Categorization	Metro	4812 (81%)	3836 (78%)	8648 (80%)	<0.001
	Urban	968 (16%)	941 (19%)	1909 (18%)	
	Rural	157 (3%)	131 (3%)	288 (3%)	
Distance from patient’s residence to hospital (miles)	<5	2125 (35%)	1429 (28%)	3554 (32%)	<0.001
	5–30	2800 (46%)	1961 (39%)	4761 (43%)	
	>30	1175 (19%)	1659 (33%)	2834 (25%)	
Charlson-Deyo Comorbidity Score	0	3942 (65%)	3119 (62%)	7061 (63%)	<0.001
	1	1475 (24%)	1399 (28%)	2874 (26%)	
	2	465 (8%)	375 (7%)	840 (8%)	
	3+	218 (4%)	156 (3%)	374 (3%)	
Diagnosis Year	2004–2010	2866 (47%)	1821 (36%)	4687 (42%)	<0.001
	2011–2017	3234 (53%)	3228 (64%)	6462 (58%)	
Primary Site	Glottis	1342 (22%)	1581 (31%)	2923 (26%)	<0.001
	Supraglottis	3065 (50%)	1631 (32%)	4696 (42%)	
	Subglottis	235 (4%)	232 (5%)	467 (4%)	
	Laryngeal Cartilage	9 (0%)	4 (0%)	13 (0%)	
	Overlapping sites of the Larynx	327 (5%)	726 (14%)	1053 (9%)	
	Larynx, NOS	1122 (18%)	875 (17%)	1997 (18%)	
Clinical nodal stage	N0	2478 (52%)	2640 (63%)	5118 (57%)	<0.001
	N1	851 (18%)	645 (15%)	1496 (17%)	
	N2	1218 (25%)	848 (20%)	2066 (23%)	
	N3	230 (5%)	49 (1%)	279 (3%)	
Lymphovascular Space Invasion	Not present	551 (89%)	2005 (63%)	2556 (67%)	<0.001
	Present	69 (11%)	1195 (37%)	1264 (33%)	
Regional lymph node surgery	No	5800 (96%)	441 (9%)	6241 (56%)	<0.001
	Yes	256 (4%)	4594 (91%)	4850 (44%)	
Primary Site Surgery	No	6014 (100%)	0 (0%)	6014 (54%)	<0.001
	Yes	0 (0%)	5049 (100%)	5049 (46%)	
Timing of radiation and surgery	No radiation and/or surgery	5880 (99%)	1520 (31%)	7400 (69%)	<0.001
	Radiation before surgery	10 (0%)	140 (3%)	150 (1%)	
	Radiation after surgery	30 (1%)	3219 (66%)	3249 (30%)	
Chemotherapy	No	1171 (22%)	2721 (61%)	3892 (40%)	<0.001
	Yes	4200 (78%)	1718 (39%)	5918 (60%)	
Tumor HPV Category	Negative	782 (13%)	781 (16%)	1563 (14%)	<0.001
	Positive	200 (3%)	147 (3%)	347 (3%)	
	Unknown	5075 (84%)	4108 (82%)	9183 (83%)	
Total Dose of Radiation (cGy)	Mean (SD)	7587 (8455)	6840 (7440.3)	7183.8 (7928.4)	0.125

SD: standard deviation; NOS: not otherwise specified; cGy: centigray.

**Table 2:** Multivariable Analysis for Total Laryngectomy Utilization.

	Variable	Odds Ratio	95% LCI	95% UCI	*p*-Value
Age	≤61 years	REF			
	>61 years	0.81	0.72	0.91	<0.001
Facility Type	Community Cancer Program	REF			
	Comprehensive Community Cancer Program	1.10	0.85	1.41	0.477
	Academic/Research Program	3.06	2.39	3.92	<0.001
	Integrated Network Cancer Program	1.50	1.15	1.96	0.003
Facility Location	New England	REF			
	Middle Atlantic	1.00	0.76	1.32	0.989
	South Atlantic	1.24	0.94	1.62	0.124
	East North Central	1.22	0.93	1.61	0.147
	East South Central	0.87	0.64	1.18	0.356
	West North Central	1.58	1.16	2.16	0.004
	West South Central	1.52	1.13	2.05	0.006
	Mountain	1.07	0.73	1.56	0.747
	Pacific	1.46	1.08	1.97	0.014
Sex	Female	REF			
	Male	1.19	1.05	1.36	0.001
Ethnicity	Non-Hispanic	REF			
	Hispanic	0.84	0.68	1.04	0.102
	Unknown	1.04	0.82	1.33	0.732
Insurance Type	Private Insurance/Managed Care	REF			
	Not Insured	0.91	0.75	1.10	0.337
	Medicaid	1.31	1.12	1.52	<0.001
	Medicare	0.97	0.84	1.11	0.628
	Other Government	0.83	0.56	1.22	0.345
	Insurance Status Unknown	0.66	0.48	0.93	0.016
Educational Attainment (Percent No High School Degree 2008–2012)	≥21.0%	REF			
	13.0–20.9%	1.01	0.88	1.16	0.897
	7.0–12.9%	1.01	0.85	1.18	0.950
	<7.0%	1.00	0.80	1.23	0.962
Income	1st Quartile	REF			
	2nd Quartile	1.13	0.97	1.32	0.110
	3rd Quartile	1.20	1.00	1.42	0.045
	4th Quartile	1.09	0.90	1.32	0.404
County Categorization	Metro	REF			
	Urban	0.83	0.70	0.97	0.019
	Rural	0.75	0.54	1.04	0.085
Distance from patient’s residence to hospital (miles)	<5	REF			
	5–30	1.35	1.19	1.53	<0.001
	>30	2.55	2.17	3.00	<0.001
Charlson-Deyo Comorbidity Score	0	REF			
	1	1.25	1.12	1.40	<0.001
	2	1.02	0.85	1.23	0.800
	3	0.74	0.57	0.97	0.031
Diagnosis Year	2004–2010	REF			
	2011–2017	1.40	1.26	1.55	<0.001
Primary Site	Glottis	REF			
	Supraglottis	0.51	0.45	0.58	<0.001
	Subglottis	0.86	0.68	1.09	0.202
	Laryngeal cartilage	0.36	0.09	1.49	0.159
	Overlapping lesions of larynx	1.99	1.65	2.40	<0.001
	Larynx, NOS	0.76	0.66	0.88	<0.001
Clinical nodal stage	N0	REF			
	N1	0.78	0.68	0.89	<0.001
	N2	0.67	0.59	0.76	<0.001
	N3	0.21	0.15	0.30	<0.001
Tumor HPV Category	Negative	REF			
	Positive	0.80	0.57	1.12	0.195
	Unknown	0.88	0.76	1.02	0.094

LCI: Lower Confidence Interval; UCI: Upper Confidence Interval; NOS: not otherwise specified.

**Table 3:** Multivariable Analysis for Survival.

	Variable	Hazard Ratio (95% CI)	*p*-Value
Facility Type	Community Cancer Program	REF	
	Comprehensive Community Cancer Program	1.05 (0.92, 1.20)	0.472
	Academic/Research Program	0.99 (0.86, 1.13)	0.825
	Integrated Network Cancer Program	1.01 (0.88, 1.17)	0.880
Age	≤61 years	REF	
	>61 years	1.32 (1.23, 1.42)	<0.001
Sex	Female	REF	
	Male	1.13 (1.05, 1.21)	0.002
Race	White	REF	
	Black	0.97 (0.90, 1.05)	0.424
	Other/unknown	0.87 (0.72, 1.05)	0.154
Ethnicity	Non-Hispanic	REF	
	Hispanic	0.88 (0.77, 1.01)	0.069
	Unknown	1.11 (0.98, 1.27)	0.110
Insurance Type	Private Insurance/Managed Care	REF	
	Not Insured	1.12 (1.00, 1.26)	0.056
	Medicaid	1.32 (1.20, 1.44)	<0.001
	Medicare	1.52 (1.40, 1.65)	<0.001
	Other Government	1.43 (1.16, 1.78)	0.001
	Insurance Status Unknown	1.26 (1.04, 1.53)	0.016
Percentage with No High School Degree	≥21.0%	REF	
	13.0–20.9%	1.04 (0.96, 1.13)	0.314
	7.0–12.9%	1.07 (0.98, 1.18)	0.124
	<7.0%	0.95 (0.84, 1.07)	0.374
Annual Income	1st Quartile	REF	
	2nd Quartile	0.99 (0.91, 1.08)	0.780
	3rd Quartile	1.03 (0.94, 1.14)	0.524
	4th Quartile	1.11 (1.00, 1.23)	0.061
Distance from patient’s residence to hospital (miles)	<5	REF	
	5–30	0.94 (0.87, 1.00)	0.061
	>30	0.97 (0.89, 1.05)	0.448
Charlson-Deyo Comorbidity Score	0	REF	
	1	1.11 (1.04, 1.19)	<0.001
	2	1.46 (1.32, 1.61)	<0.001
	3+	1.41 (1.21, 1.64)	<0.001
Diagnosis Year	2004–2010	REF	
	2011–2017	0.94 (0.88, 1.00)	0.049
Primary Site	Glottis	REF	
	Supraglottis	1.09 (1.01, 1.17)	0.031
	Subglottis	1.15 (1.00, 1.32)	0.058
	Laryngeal cartilage	0.97 (0.43, 2.16)	0.933
	Overlapping lesions of larynx	1.16 (1.04, 1.29)	0.009
	Larynx, NOS	1.29 (1.19, 1.40)	<0.001
Clinical nodal stage	N0	REF	
	N1	1.10 (1.02, 1.20)	0.014
	N2	1.41 (1.31, 1.51)	<0.001
	N3	1.77 (1.52, 2.05)	<0.001
Total Laryngectomy	No	REF	
	Yes	0.50 (0.47, 0.54)	<0.001
Radiation	No	REF	
	Yes	0.49 (0.46, 0.53)	<0.001
Chemotherapy	No	REF	
	Yes	0.94 (0.87, 1.01)	0.079
Tumor HPV Status	Negative	REF	
	Positive	0.68 (0.53, 0.86)	0.002
	Unknown	1.00 (0.91, 1.10)	0.962

CI: Confidence Interval; NOS: not otherwise specified.

## Data Availability

The data utilized in this study are available via an application process to investigators associated with CoC-accredited cancer programs.

## Supplementary Materials

The following supporting information can be downloaded at: https://www.mdpi.com/article/10.3390/cancers15225447/s1, Supplemental Table S1: univariate analysis for total laryngectomy utilization; Supplemental Table S2: reason for no surgery among patients not receiving total laryngectomy; Supplemental Table S3: univariate analysis for survival.
