# Supplementary material for: Factors Associated with Total Laryngectomy Utilization in Patients with cT4a Laryngeal Cancer
Source: Cancers (Basel). 2023 Nov 16;15(22):5447. doi: 10.3390/cancers15225447 (PMC10670908; doi:10.3390/cancers15225447)
Supplement: Supplementary file 1 [file cancers-15-05447-s001.zip › Supplemental Table S1 (Univariate Results Surgery).pdf]

Supplemental Table S1. Univariate Analysis for Total Laryngectomy Utilization

LCI: Lower Confidence Interval; UCI: Upper Confidence Interval; NOS: Not Otherwise Specified

	Variable	Odds Ratio	95% LCI	95% UCI	P-value
**Age**	≤61 years	**REF**			
	>61 years	0.78	0.73	0.84	<0.001
**Facility Type**	Community Cancer Program	**REF**			
	Comprehensive Community Cancer Program	1.17	0.95	1.44	<0.001
	Academic/Research Program	3.38	2.77	4.12	<0.001
	Integrated Network Cancer Program	1.53	1.23	1.89	0.587
**Facility Location**	New England	**REF**			
	Middle Atlantic	1.26	1.02	1.55	0.018
	South Atlantic	1.44	1.18	1.76	0.643
	East North Central	1.49	1.22	1.83	0.232
	East South Central	1.16	0.93	1.45	0.001
	West North Central	1.87	1.49	2.35	<0.001
	West South Central	1.84	1.48	2.30	<0.001
	Mountain	1.18	0.89	1.56	0.063
	Pacific	1.78	1.41	2.24	<0.001
**Sex**	Female	**REF**			
	Male	1.29	1.17	1.41	<0.001
**Race**	White	**REF**			
	Black	0.94	0.85	1.03	0.955
	Other/Unknown	0.88	0.69	1.12	0.449
**Ethnicity**	Non-Hispanic	**REF**			
	Hispanic	0.98	0.83	1.14	0.099
	Unknown	0.70	0.58	0.85	0.001
**Insurance Type**	Private Insurance/Managed Care	**REF**			
	Not Insured	0.92	0.80	1.06	0.909
	Medicaid	1.28	1.15	1.43	<0.001
	Medicare	0.87	0.79	0.96	0.143
	Other Government	0.83	0.64	1.09	0.367
	Insurance Status Unknown	0.73	0.56	0.96	0.039
**Percentage with No High School Degree**	≥21.0%	**REF**			
	13.0% - 20.9%	1.00	0.91	1.11	0.100
	7.0%-12.9%	0.93	0.84	1.04	0.568
	<7.0%	0.87	0.77	1.00	0.059
**Income**	1st Quartile	**REF**			
	2nd Quartile	1.13	1.01	1.26	0.481

	3rd Quartile	1.21	1.08	1.35	0.011
	4th Quartile	1.08	0.97	1.21	0.624
**County Categorization**	Metro	**REF**			
	Urban	1.22	1.10	1.35	0.020
	Rural	1.05	0.83	1.33	0.658
**Distance from patient's residence to hospital (miles)**	<5	**REF**			
	5-30	1.04	0.95	1.14	<0.001
	>30	2.10	1.90	2.32	<0.001
**Charlson-Deyo Comorbidity Score**	0	**REF**			
	1	1.20	1.10	1.31	<0.001
	2	1.02	0.88	1.18	0.920
	3+	0.90	0.73	1.12	0.122
**Diagnosis Year**	2004-2010	**REF**			
	2011-2017	1.57	1.46	1.70	<0.001
**Primary Site**	Glottis	**REF**			
	Supraglottis	0.45	0.41	0.50	<0.001
	Subglottis	0.84	0.69	1.02	0.384
	Laryngeal cartilage	0.38	0.12	1.23	0.170
	Overlapping lesions of larynx	1.88	1.62	2.19	<0.001
	Larynx, NOS	0.66	0.59	0.74	0.251
**Clinical nodal stage**	N0				
	N1	0.71	0.63	0.80	<0.001
	N2	0.65	0.59	0.73	0.001
	N3	0.20	0.15	0.27	<0.001
**Tumor HPV Category**	Negative	**REF**			
	Positive	0.74	0.58	0.93	0.072
	Unknown	0.81	0.73	0.90	0.370
