# Supplementary material for: Factors Associated with Total Laryngectomy Utilization in Patients with cT4a Laryngeal Cancer
Source: Cancers (Basel). 2023 Nov 16;15(22):5447. doi: 10.3390/cancers15225447 (PMC10670908; doi:10.3390/cancers15225447)
Supplement: Supplementary file 1 [file cancers-15-05447-s001.zip › Supplemental Table S2 (Reasons for No Surgery).pdf]

Supplemental Table S2. Reason for no surgery among patients not receiving total laryngectomy

Reason for No Surgery	Frequency	Percentage
Not recommended as part of treatment	5088	85.7%
Contraindicated due to risk factors	296	5.0%
Patient died prior to surgery	45	0.8%
Recommended but not performed (reason not documented in chart)	62	1.0%
Recommended but refused by patient, family member, or guardian	448	7.5%
**Total**	**5939**	
