# Supplementary material for: Factors Associated with Total Laryngectomy Utilization in Patients with cT4a Laryngeal Cancer
Source: Cancers (Basel). 2023 Nov 16;15(22):5447. doi: 10.3390/cancers15225447 (PMC10670908; doi:10.3390/cancers15225447)
Supplement: Supplementary file 1 [file cancers-15-05447-s001.zip › Supplemental Table S3 (Univariate Results Survival).pdf]

**Supplemental Table S3. Univariate Analysis for Survival**

CI: Confidence Interval; NOS: Not Otherwise Specified

| | | Hazard Ratio (95% CI) | p-value |
|---|---|---|---|
| **Facility Type** | Community Cancer Program | **REF** | |
| | Comprehensive Community Cancer Program | 0.99 (0.88, 1.10) | 0.798 |
| | Academic/Research Program | 0.81 (0.72, 0.90) | <0.001 |
| | Integrated Network Cancer Program | 0.96 (0.85, 1.08) | 0.507 |
| **Facility Location** | New England | **REF** | |
| | Middle Atlantic | 0.91 (0.80, 1.02) | 0.110 |
| | South Atlantic | 0.96 (0.85, 1.08) | 0.457 |
| | East North Central | 0.88 (0.78, 0.99) | 0.029 |
| | East South Central | 1.0 (0.88, 1.14) | 0.985 |
| | West North Central | 0.9 (0.78, 1.03) | 0.118 |
| | West South Central | 0.86 (0.75, 0.98) | 0.024 |
| | Mountain | 0.91 (0.77, 1.08) | 0.301 |
| | Pacific | 0.89 (0.78, 1.02) | 0.101 |
| **Age** | ≤61 years | **REF** | |
| | >61 years | 1.60 (1.53, 1.68) | <0.001 |
| **Sex** | Female | **REF** | |
| | Male | 1.09 (1.02, 1.15) | 0.006 |
| **Race** | White | **REF** | |
| | Black | 1.08 (1.02, 1.15) | 0.006 |
| | Other/Unknown | 0.88 (0.75, 1.03) | 0.113 |
| **Ethnicity** | Non-Hispanic | **REF** | |
| | Hispanic | 0.82 (0.73, 0.91) | <0.001 |
| | Unknown | 1.21 (1.09, 1.35) | <0.001 |
| **Insurance Type** | Private Insurance/Managed Care | **REF** | |
| | Not Insured | 1.14 (1.04, 1.25) | 0.007 |
| | Medicaid | 1.19 (1.11, 1.28) | <0.001 |
| | Medicare | 1.82 (1.71, 1.93) | <0.001 |
| | Other Government | 1.51 (1.28, 1.78) | <0.001 |
| | Insurance Status Unknown | 1.43 (1.21, 1.68) | <0.001 |
| **Percentage with No High School Degree** | ≥21.0% | **REF** | |
| | 13.0% - 20.9% | 1.03 (0.97, 1.10) | 0.328 |
| | 7.0%-12.9% | 1.06 (1.00, 1.13) | 0.062 |
| | <7.0% | 0.96 (0.89, 1.04) | 0.360 |
| **Income** | 1st Quartile | **REF** | |
| | 2nd Quartile | 1.04 (0.97, 1.11) | 0.265 |
| | 3rd Quartile | 1.04 (0.97, 1.11) | 0.299 |
| | 4th Quartile | 1.07 (1.00, 1.15) | 0.048 |
| **County Categorization** | Metro | **REF** | |

| | | | |
|---|---|---|---|
| | Urban | 1.02 (0.96, 1.09) | 0.438 |
| | Rural | 1.10 (0.96, 1.27) | 0.163 |
| **Distance from patient's residence to hospital (miles)** | <5 | **REF** | |
| | 5-30 | 1.05 (0.99, 1.11) | 0.064 |
| | >30 | 1.02 (0.96, 1.09) | 0.470 |
| **Charlson-Deyo Comorbidity Score** | 0 | **REF** | |
| | 1 | 1.13 (1.07, 1.19) | <0.001 |
| | 2 | 1.51 (1.39, 1.65) | <0.001 |
| | 3 | 1.76 (1.56, 1.99) | <0.001 |
| **Diagnosis Year** | 2004-2010 | **REF** | |
| | 2011-2017 | 0.87 (0.83, 0.91) | <0.001 |
| **Primary Site** | Glottis | **REF** | |
| | Supraglottis | 1.19 (1.13, 1.27) | <0.001 |
| | Subglottis | 1.14 (1.01, 1.29) | 0.037 |
| | Laryngeal cartilage | 1.37 (0.74, 2.55) | 0.319 |
| | Overlapping lesions of larynx | 1.13 (1.03, 1.23) | 0.010 |
| | Larynx, NOS | 1.36 (1.27, 1.46) | <0.001 |
| **Clinical nodal stage** | N0 | **REF** | |
| | N1 | 1.08 (1.01, 1.16) | 0.030 |
| | N2 | 1.33 (1.25, 1.42) | <0.001 |
| | N3 | 1.92 (1.67, 2.19) | <0.001 |
| **Total Laryngectomy** | No | **REF** | |
| | Yes | 0.58 (0.55, 0.60) | <0.001 |
| **Radiation** | No | **REF** | |
| | Yes | 0.56 (0.53, 0.59) | <0.001 |
| **Chemotherapy** | No | **REF** | |
| | Yes | 1.09 (1.04, 1.15) | <0.001 |
| **Lymphovascular Space Invasion** | Not Present | **REF** | |
| | Present | 1.43 (1.30, 1.57) | <0.001 |
| **Tumor HPV Status** | | | |
| | Negative | **REF** | |
| | Positive | 0.81 (0.68, 0.96) | 0.016 |
| | Unknown | 1.08 (1.00, 1.16) | 0.030 |
